# Validation of an interviewer-administered seven-day semi-quantitative food frequency questionnaire for the dietary assessment of preschool children in rural Bangladesh

**DOI:** 10.1017/jns.2021.19

**Published:** 2021-04-19

**Authors:** Sabuktagin Rahman, Patricia Lee, Santhia Ireen, Moudud ur-Rahman Khan, Faruk Ahmed

**Affiliations:** 1 Public Health, School of Medicine, Griffith University, Building GO1, Gold Coast Campus, Southport, QLD 4222, Australia; 2 Alive and Thrive, FHI 360, Bangladesh Country Office, Dhaka 1212, Bangladesh; 3 Institute of Nutrition and Food Science, University of Dhaka, Dhaka 1000, Bangladesh

**Keywords:** Bangladesh, Dietary assessment, Food frequency questionnaire, Preschool children

## Abstract

A validation study of an interviewer-administered, seven-day semi-quantitative food frequency questionnaire (7-d SQFFQ) was conducted in Bangladeshi rural preschool age children. Using a cross-sectional study design, 105 children from 103 households were randomly selected. For the SQFFQ, a list of commonly consumed foods was adapted from the Bangladesh national micronutrient survey 2011–12. The data on the actual number of times and the amount of the children's consumption of the foods in the preceding 1 week were collected by interviewing the mothers. The intake was compared with two non-consecutive days 24-h dietary recalls conducted within 2 weeks after the SQFFQ. Validity was assessed by the standard statistical tests. After adjusting for the energy intake and de-attenuation for within-subject variation, the food groups (cereals, animal source foods, milk and the processed foods) had ‘good’ correlations between the methods (rho 0⋅65–0⋅93; *P* < 0⋅001). Similarly, the macronutrients (carbohydrate, protein and fats) had ‘good’ correlations (rho 0⋅50–0⋅75; *P* < 0⋅001) and the key micronutrients (iron, zinc, calcium, vitamin A, etc.) demonstrated ‘good’ correlations (rho 0⋅46–0⋅85; *P* < 0⋅001). The variation in classifying the two extreme quintiles by the SQFFQ and the 24-h recalls was <10 %. The results from Lin's concordance coefficients showed a ‘moderate’ to ‘excellent’ absolute agreement between the two methods for food groups, and nutrients (0⋅21–0⋅90; *P* < 0⋅001). This interviewer-administered, 7-d SQFFQ with an open-ended intake frequency demonstrated adequate validity to assess the dietary intake for most nutrients and suitable for dietary assessments of young children in Bangladesh.

## Introduction

Childhood is an important period in the life cycle because it is a phase of intense growth and development. The nutritional needs during childhood are increased significantly, and thus, adequate nutrition and dietary intake are essential^(1)^. The dietary assessment tool has been used to establish the relationship of population's eating habits with the presence of morbidity and mortality, allowing early detection of nutritional deficiencies in vulnerable groups, such as children^(2)^. A food frequency questionnaire (FFQ) is one of the most commonly used dietary assessment tools in nutritional epidemiological studies and surveys^(3)^. The FFQ consists of a predetermined list of foods and beverages with response categories to indicate the usual frequency of consumption over a specified period^(4)^.

The advantages of FFQ are easier to administer, usually less time consuming to implement, captures individual-level dietary patterns and better at estimating ‘usual diet’ due to longer recall^(5)^. Traditionally, the FFQ is respondent-administered and designed with a close-ended frequency option for the consumption of various food items. However, the self-reported FFQ can lead to some measurement error due to within-subject variability, lack of ability to report food consumption and difficulties in recalling which and how much food was consumed^(6)^. In rural Bangladesh, the majority of the respondents are functionally illiterate, and thus, the respondent-administered FFQ may introduce a bias while assessing the food and nutrient intakes. To ameliorate these respondent issues, recent epidemiological dietary assessments in Bangladesh used an interviewer-administered, open-ended intake-response seven-day semi-quantitative FFQ (7-d SQFFQ)^(7,8)^. To date, the FFQ has not been validated. Furthermore, the close-ended frequency options of consumption in the traditional FFQ has an inherent limitation, as the respondent's actual consumption often does not match the specified consumption categories in the questionnaire^(9)^. Traditionally, FFQs inquire about the frequency of consumption over a long time (up to a year) and provide information on the habitual frequency of dietary intake of individuals, instead of an actual number of times of consumption over the reference period. Hence, some inaccuracies are expected regarding the amount of consumption.

There is a paucity of FFQ validation in Bangladesh. Lin *et al.*^(10)^ validated a dish-based FFQ with two 3-d food-records in a mixed population group consisting of children and adults (median age 30 years). Though the dish-based FFQ is contextually relevant in Bangladesh, the lack of specificities of food items is likely to impart a difference in the actual nutrient intake as the possible different foods within a dish might vary considerably in nutrient content. Additionally, consistent with any FFQ, the study included the wide frequency-categories of intake which might put a respondent confused as his/her intake might not belong to any of the categories.

Chen *et al.*^(11)^ validated the other prominent FFQ with the Bangladeshi traditional diet in adult male and female subjects. This consisted of commonly consumed foods (thirty-nine items) in the rural setting of Bangladesh and compared the FFQ with two 7-d Food Diary (FD). Consistent with the proposed SQFFQ, Chen *et al.* employed the interviewer-administered FFQ and kept an open-ended frequency option. However, the major limitation was that the Food Composition Tables (FCTs) used were either the USDA database^(12)^ or the FCT of the neighbouring India^(13)^. Usage of the extraneous FCTs unlikely to reflect the most accurate nutrient values of locally produced food, as food composition varies from country to country depending on the species of plants and animals, agricultural technology, climatic condition, processing and storage circumstances^(14)^.

Taking into consideration of the above issues, we conducted the present study to assess the validity of an interviewer-administered 7-d SQFFQ designed to measure food and nutrient intake, with a particular interest on micronutrient intakes, and to be used in a community-based trial examining the efficacy of a low-iron micronutrient powder (MNP) in preschool children in rural Bangladesh. The distinguishing features of this SQFFQ are a short reference time (1 week) and the open-ended frequency of intake option, i.e. the actual number of times of consumption.

## Methods

### Participants

The present study was conducted on 105 children, aged 24–59 months, recruited from 103 households in Belkuchi, a rural sub-district in a north-central district of Bangladesh. The participants were recruited using simple random sampling. The field staff identified the households with children of the stipulated age by a door-to-door visit. The purpose and exact nature of the study were explained to all eligible mothers or the primary caretakers of the children, and those who agreed to participate either signed or put a thumb impression on the consent form. The study was nested in a trial examining the efficacy of a low-iron micronutrient formulation in children of rural Bangladesh. The trial was approved by the research ethics committees of the University of Dhaka, Bangladesh (Ref# 46/Biol. Scs. /2017–2018) and Griffith University, Australia (Ref# 2017/467).

### Study design

There is no definitive ‘gold standard’ in dietary assessment, nor is there a ‘gold standard’ for assessing the validity of FFQ^(9)^. Therefore, the estimation of a tool's relative validity relies upon a comparison with a superior and preferably independent technique, known as comparative validation^(15)^. For a reference method, both weighed food record (WFR) and 24-h dietary recall (DR) are commonly used due to their greater precision in the quantification of intake^(15)^. WFR is a suitable candidate for FFQ validation – but the need for good literacy and numeracy precludes its use in the rural Bangladesh context. Biochemical methods as the reference method to validate the SQFFQ, although less prone to errors involved with misreporting or poor memory, are expensive, invasive and nutrient-specific and, hence, not considered in the present validation study^(9)^. Considering the low literacy level of the respondents, 24-h DR was chosen as the reference method.

**Fig. 1.**
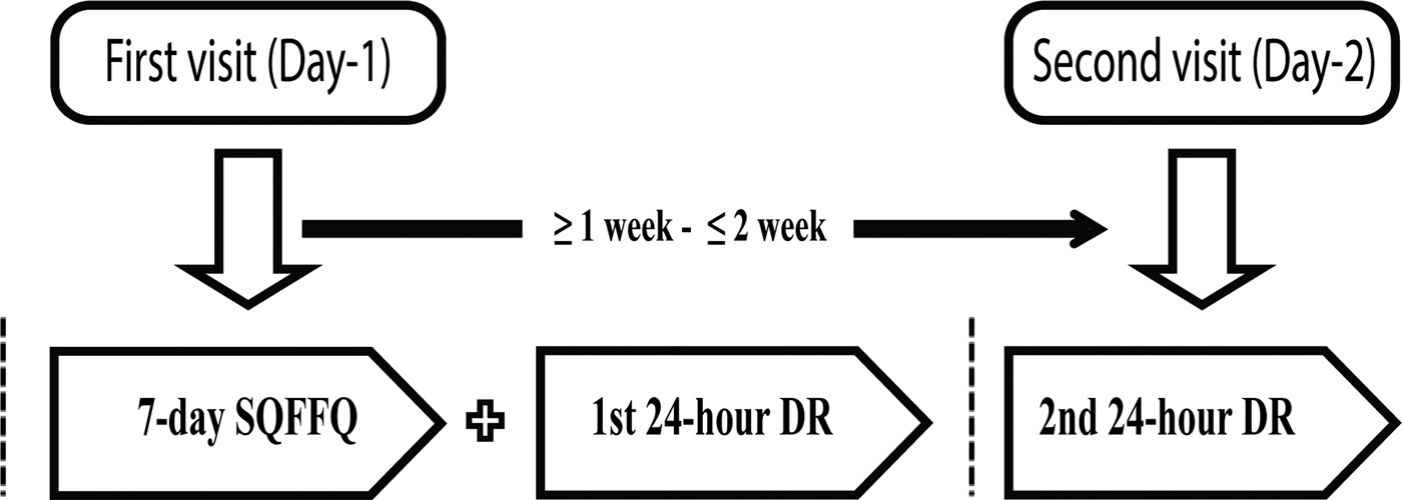
Design of the 7-d SQFFQ validation study.

The children's food intake was measured by interviewing the mothers or caregivers, using a 7-d SQFFQ which was adopted from a national survey and a study in Bangladesh^(7,8)^. The validity of the nutrient intake measured by the SQFFQ was assessed by comparing to the average intake of the two 24-h DRs as the reference method, administered on non-consecutive weekdays. The interval between the 24-h DRs was ≥1 to ≤2 weeks (Fig. 1).

The respondents (mother or the primary caretaker of the child) were visited twice. On the first visit, first, the SQFFQ was administered, followed by the first 24-h DR. During the second visit, after 1 week but within 2 weeks of the first visit, the second 24-h DR was conducted. Data were collected by the trained interviewers. One of the researchers monitored the data collection to ensure the quality of data.

### Sample size

A total of 105 participants were included in the study. Bland–Altman plot is one of the most used statistical techniques to assess agreement in dietary validation studies. The sample size was considered on a recommendation that a minimum of 50 subjects is required if the Bland–Altman statistics are to be estimated, with a suggestion of 100 for the study^(9)^.

### Development of the SQFFQ

#### Selection of foods

The FFQ was adopted from the Bangladesh national micronutrient survey 2011–12^(16)^ and a recent dietary intake assessment study^(8)^, and pretested in the study population. The food list considered in the national micronutrient survey was referred from the comprehensive food consumption survey – a nationally representative dietary study^(17)^. It is important to note that the dietary habit of the Bangladeshi population is grossly homogenous with little diversity. The principles of the selection of the foods were as follows:

1. Foods most commonly consumed in the Bangladeshi population.
2. Foods rich in a particular nutrient. For example, several leafy vegetables were considered, because the foods are universally consumed across the population and it is the largest source of a number of micronutrients in the setting.

Furthermore, the processed foods were added to the list of the foods. The consumption of processed foods among children has increased over the last decade. We referred to the pretested list of the processed food from the study of Iqbal *et al.*^(8)^. The processed foods are ready-to-eat, locally produced, energy-rich, fatty and sugary with poor content of nutrients.

The SQFFQ used a total of fifty-three commonly consumed foods in the rural setting of Bangladesh. These foods were grouped as cereals, legumes, leafy and non-leafy vegetables, yellow/orange vegetables, fruits, small fishes, large fishes, meats, eggs, organ meats and some ready-to-eat processed foods. Among the cereals were rice, hand-made flat bread, sliced bread, oil-fried bread and puffed rice. Meats included were chicken, beef, goat and liver. Fishes included small fishes which are eaten whole along with bones; and the most commonly eaten large fishes, e.g. carps and catfishes. Fruits included commonly eaten indigenous fruits, e.g. mangoes, jackfruits, ripe banana, guava, plums and few others. The imported fruits included oranges, malta, apples and pomegranate. Non-leafy vegetables included commonly consumed items: sweet pumpkin, potatoes and gourds. The processed foods included were commonly preferred by the children: cakes, sweet biscuits, candies, juice drinks, chocolates, fried flour-made snacks and few others. Portion size was one serving amount. We used the portion size reference of the Institute of Nutrition and Food Science, University of Dhaka, which describes the serving size of the commonly eaten local foods^(18)^.

#### Development of the food album

The food album was intended to assist the respondents (i.e. mother) in assessing the amount of a particular food consumed by the children. It contained the principal foods listed in the SQFFQ. To develop the food album, for the cooked food we weighed the foods to a 1-g precision by a kitchen scale (SECA 852 digital diet scale) and kept in the plates/bowl in an amount of its serving size. The photos of the foods were captured in a standardised way, i.e. at the same angle, at the same distance, and placed on a standard plate/bowl to standardise the relative size as it would appear when looked at. The raw food items and the ready-to-eat processed foods were photographed directly.

#### Conduction of the SQFFQ

The mother of the child was asked about the food intakes of her child as per the following guidelines:

1. Did the child consume a particular food (i.e. listed food) in the preceding 1 week?
2. How many days in the last week did the child consume the food?
3. How many times each day did the child consume the food?
4. How much food (on average) each time did the child consume?

From questions (2) and (3), the information derived was on the absolute number of times that the particular food was taken over the week. From question (4), information on the average amount of intake each time the food was consumed was gathered. The food album displayed the food items to their serving amount, e.g. one half-plate full of leafy vegetables displayed amounting to its one serving. The enumerators explored the average amount of intake of a particular food by proportioning the amount displayed. In this way, they calculated both the serving amount and absolute amount (in grams/millilitres) taken over the week. The amount of the intake was also assessed by displaying standardised bowls, glasses and spoons. These containers were pre-standardised by loading with the commonly eaten local food items, ink-marked at various levels and weighed by an electronic scale with 1 g resolution (Seca Culina 852). At the interview, how much of the displayed container-load of the food the child consumed was inquired to the mother, and the amount was recorded. In the case of liquid foods, e.g. pulses, the measurement was done by asking the mother to pour plain water into the supplied graduated measuring beaker with 1 ml resolution from the bowls/glasses she used to feed the child. The amount was recorded in millilitres. The reported amount of weekly food consumption data was converted to the daily average intake by dividing by 7 (seven). For processed foods that were purchased, the inquiry was made into the brand names, how much money spent to purchase, and availability of the empty packets, to gather the information on the amount of the portion.

### 24-h DR

For the 24-h DRs, the preceding 24 h was segregated into six time periods: breakfast, mid-morning, lunch, afternoon snacks, dinner and bedtime. The amount of consumption of all food items over the period was assessed by the interviewers. The amount of consumption by the 24-h DRs was measured following the same principle used for the SQFFQ. Weekends and special days, such as festivals and mourning, were not considered for the 24-h DRs.

### Nutrient estimation

An updated FCT on Bangladeshi foods was used to calculate the nutrient intakes^(19)^. For a few nutrients which were missing in the FCT, the USDA database on the nutrient values was used^(20)^. The edible portion coefficients for Bangladeshi foods were used to derive the edible amount^(19)^. The cooked-food amounts were converted into the raw food weight, by dividing with the appropriate yield factors^(19)^. The nutrient values were calculated per 100 g of the raw weight of the consumption as per the indication in the FCTs. For the non-cooked foods or the ready-to-eat processed foods, the nutrient values were directly gathered from the FCTs. For processed foods, information was also gathered from the nutrient facts labelled on the packets.

### Statistical analysis

The food and nutrients intake data from the SQFFQ and the average of two 24-h DRs were tested for normality. Based on the Shapiro–Wilk testing (results not shown), the distribution of energy and nutrients intake were not reporting the normal distribution. Thus, mean (sd) and median with interquartile ranges (IQRs) were estimated for energy and nutrient intakes for the test and the reference methods.

We compared the food and nutrients intake data between the SQFFQ and 24-h DRs using the Wilcoxon signed-rank test. Spearman rank correlation coefficient (rho) was used to assess the strength and direction of the association between food and nutrients intakes measured by the SQFFQ and 24-h DRs.

The reference method (i.e. 24-h DRs) can be imperfect and subject to within-person variation and/or day-to-day deviations, leading to the underestimated measures, i.e. the correlation coefficient underestimating the degree of agreement. This underestimation is known as ‘attenuation bias’^(21)^. To minimise the attenuation bias, we computed the energy-adjusted correlation of the food and nutrients intakes obtained from the two methods. To adjust for energy intake, the nutrient density was calculated by dividing the mean nutrient value by the mean energy intake. The estimate was used in the Spearman rank correlational analysis instead of the original value of nutrient intake as recommended by Bingham *et al.*^(22)^. Furthermore, since the random within-individual variation in the measurement of any of the variables being compared tends to reduce correlation coefficients towards zero^(23,24)^, correlations with corrections for the attenuated effects of such measurement error in the two 24-h DRs are calculated, by using the following formula:
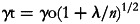
where *γ*t is the true correlation coefficient; *γ*o is the observed energy-adjusted correlation coefficient of the intakes recorded by the methods; *λ* is the ratio of the within-individual to between-individual variances of the daily intakes and *n* is the number of replicates (here, *n* 2 as two 24-h DRs were administered).

To calculate *λ*, one-way analysis of variance (ANOVA) of foods and nutrients intakes measured by the two 24-h DRs was computed to yield the variances (results not shown). The mean percentage differences of all food groups and nutrients intake between the test and reference methods were used to assess the agreement at the group level (size and direction of error)^(25,26)^. For the calculation of the mean percentage difference, the reference value was subtracted from the test measure value, divided by the reference measure and multiplied by 100 for each participant^(27)^. Furthermore, SQFFQ's ability to rank the consumption correctly was examined by the cross-quartile classification analysis. Participants whose intakes were ranked by the SQFFQ to the opposite extreme quintile of intakes as per their responses in the 24-h DRs were considered grossly misclassified. The proportion of the measurements by both the methods falling in the same quintile was calculated, though the agreement may occur by chance^(28)^.

To assess the extent of the agreement by accounting for chance, we used the weighted kappa statistic (*w*) with pre-recorded weights, which assessed the inter-rater agreement of the measures estimated by the two methods while accounting for the possibility of the agreement occurring by chance^(29)^. The coefficient of Lin's absolute agreement was estimated, which quantified the agreement of the two measurements of the same variable, i.e. nutrient intakes^(30)^. Lin's coefficient which measured both the precision and the accuracy of the relationship between the methods has evaluated whether the observed data deviate significantly from the line of perfect concordance^(31)^.

Bland–Altman plots were used to illustrate the agreement between the measurements (test − reference measure) (*y*-axis) against the mean of the two measures (test measure + reference measure/2) (*x*-axis) and identify the outliers and trends in bias for each subject^(27,28,32)^. The limits of agreements were estimated by using the mean and the standard deviation (sd) of the differences between the two measurements (mean difference (1⋅96 sd))^(33,34)^. Since the histograms were not perfectly bell-shaped, log-transformation was done before the testing. Data analyses were done in STATA 14⋅0 (STATA Inc., College Station, TX, USA).

### Interpretation of statistical outcomes

A number of statistical tests were performed to provide a comprehensive assessment of the various aspects of validity. A correlation coefficient ≥0⋅50 was considered ‘good’; 0⋅20–0⋅49 was ‘acceptable’, while <0⋅20 was ‘poor’^(35,36)^. The percent difference of 0–10⋅9 % was considered ‘good’, between 11 and 20 % was considered ‘acceptable’ and >20 % was ‘poor’^(26)^. Cross-classification with ≤10 % in opposite quintile was considered ‘good’ and >10 % was ‘poor’^(35)^. The weighted kappa statistics of 0⋅8–1⋅0 was considered ‘very good’, 0⋅6–0⋅8 was ‘good’, 0⋅4–0⋅6 was ‘moderate’, 0⋅2–0⋅4 was ‘fair’ and <0⋅2 was ‘poor’^(37)^. Lin's concordance coefficient <0⋅20 was considered ‘poor’, 0⋅20–0⋅80 was ‘acceptable’ and >0⋅80 was ‘excellent’^(37)^.

The quality of the present validation study was evaluated as per the guidelines of the European Micronutrient Recommendations Aligned Network of Excellence (EURRECA)^(38)^. The assessment was made on (a) the sample and sample size; (b) statistics: group means, correlations and agreements; (c) the data collection method; (d) seasonality and (e) the inclusion of supplements.

## Results

### General characteristics

Table 1 presents the socio-demographic characteristics of the study participants. The proportion of male children was 45⋅7 %. On average, the children were 37⋅3 ± 0⋅9 months old. Mothers completed on average 7⋅7 ± 0⋅3 years of schooling. ‘Improved’ (built with cement and/or corrugated iron sheet) households according to the materials used for the construction was possessed by 36⋅1 % of the respondents. Nearly half of the households (46⋅6 %) reported having their own cultivable lands. On average, BDT 1823⋅5 ± 0953⋅1 (US$ 21⋅7 ± 11⋅3) was spent for purchasing food in the week preceding the interview.

**Table 1.** Some selected socio-demographics characteristics of the study participants (*n* 105)

Traits	%
Age of child (month)	
Mean	37⋅3
sd	0⋅9
Sex (male)	45⋅7
Mother's education (years)	
Mean	7⋅7
sd	0⋅3
Improved house^a^	36⋅1
Possession of cultivable land	46⋅6
Possession of cultivable land (decimals)	
Mean	39⋅5
sd	70⋅5
Last week's spend on food^b^ (BDT)^c^	
Mean	1823⋅5
sd	953⋅1

a Semi-pacca house (Floor: cement and bricks; walls and roof: corrugated iron sheet) and pacca house (whole parts cement and bricks built).

b Rice, flour, oil, fish, meat, eggs, vegetables, etc.

c USD 21⋅7 ± 11⋅3.

### Validity of the 7-d SQFFQ

#### Comparative profile of the intakes

Table 2 shows the estimates of the daily food and nutrient intakes measured by the 7-d SQFFQ and the reference method (24-h DRs). The average of the two 24-h DRs was computed as the reference value. By using the SQFFQ, the daily intakes of the cereals, animal source foods, milk and legumes appeared higher than that measured by the average of the two 24-h DRs. Table 3 shows that the intakes were significantly higher (*P* < 0⋅05) for the food groups, measured by the SQFFQ compared with the average of two 24-h DRs, except for the legumes (*P* = 0⋅11) and the processed foods (*P* = 0⋅15).

**Table 2.** Profile of intake estimates of food and nutrients in Bangladeshi children 24–59 months old as measured by the 7-d SQFFQ and 24-h DRs

	Distribution of daily intakes							
Food/Nutrients	7-d SQFFQ				Average of two dietary recalls			
	Mean	sd	Median	IQR	Mean	sd	Median	IQR
Food groups								
Cereals^a^ (g)	128⋅2	74⋅8	116⋅7	75⋅1, 160⋅6	107⋅5	63⋅8	98⋅7	62⋅9, 138⋅4
ASF^a^ (g)	45⋅4	37⋅4	33⋅8	20⋅4,62⋅2	39⋅8	33⋅2	33⋅9	17⋅3, 57⋅1
Milk^a^ (ml)	350⋅2	418⋅2	111⋅6	15⋅6, 781⋅2	307⋅9	377⋅2	129⋅7	0, 558⋅6
Legumes^a^ (g)	6⋅4	12⋅7	2⋅8	0, 5⋅5	5⋅1	11⋅1	0⋅0	0, 5⋅3
Fruits^a^ (g)	48⋅6	65⋅2	28⋅5	5⋅3, 68⋅1	32⋅3	58⋅1	0⋅0	0, 37⋅0
Vegetables^a^ (g)	31⋅5	43⋅2	14⋅4	2⋅7, 42⋅1	22⋅3	49⋅4	5⋅1	0, 30⋅3
Processed food (g)	25⋅5	23⋅8	18⋅5	6⋅4, 37⋅2	23⋅4	26⋅7	14⋅0	5⋅0, 32⋅5
Nutrients								
Energy (kcal)	998⋅4	385⋅6	950⋅1	689⋅5, 1259⋅4	875⋅9	321⋅7	910⋅6	601⋅2, 1121⋅7
Carbohydrate (g)	157⋅2	60⋅2	148⋅1	119⋅6, 198⋅6	130⋅6	52⋅6	122⋅0	94⋅9, 163⋅9
Protein (g)	32⋅1	14⋅8	30⋅0	19⋅5, 41⋅1	27⋅2	11⋅6	28⋅4	17⋅6, 34⋅7
Fats (g)	23⋅5	17⋅0	19⋅7	11⋅1, 33⋅7	24⋅0	12⋅5	23⋅2	14⋅7, 31⋅1
Dietary fibre (g)	7⋅98	4⋅3	6⋅97	4⋅8, 10⋅2	6⋅27	3⋅9	5⋅43	3⋅63, 7⋅7
Iron (mg)	3⋅57	1⋅9	3⋅11	2⋅3, 4⋅3	2⋅87	1⋅7	2⋅39	1⋅9, 3⋅3
Zinc (mg)	5⋅3	2⋅3	5⋅1	3⋅5, 6⋅8	4⋅4	1⋅9	4⋅3	2⋅8, 5⋅7
Calcium (mg)	433⋅2	428⋅5	238⋅1	95⋅7, 827⋅4	369⋅3	378⋅5	191⋅5	67⋅9, 658⋅1
Magnesium (mg)	154⋅2	68⋅1	153⋅5	105⋅6, 195⋅6	124⋅6	54⋅1	116⋅8	81⋅1, 165⋅2
Vitamin A (μg)	299⋅2	255⋅1	245⋅2	89⋅7, 417⋅1	258⋅8	336⋅7	146⋅7	53⋅9, 327⋅5
Thiamine (mg)	0⋅67	0⋅29	0⋅66	0⋅44, 0⋅85	0⋅55	0⋅23	0⋅54	0⋅37, 0⋅73
Folates (mcg)	101⋅1	60⋅0	89⋅6	62⋅1, 129⋅9	82⋅8	65⋅2	65⋅8	40⋅8, 102⋅3
Vitamin C	32⋅6	36⋅4	23⋅1	10⋅7, 27⋅1	24⋅8	42⋅9	12⋅8	5⋅6, 27⋅1

a Raw-food weight.

**Table 3.** Results of the statistical tests for assessing the validity of the 7-d SQFFQ and the interpretation of agreement for food, energy and nutrient intakes in Bangladeshi children 24–59 months old

Food/Nutrients	Unadjusted Spearman Rank correlation coefficient (rho)*	Energy-adjusted Spearman Rank correlation coefficient*	De-attenuated correlation coefficient^a^	Wilcoxon Sign-ranked test^b^ (*P* value)	Percent difference (%)^c^	Cross-classification^d^		Weighted kappa statistics^e^	Lin's concordance coefficient of absolute agreement^f^ (rho_c)
						Same quintile (%)	Extreme quintile (%)		
Food groups									
Cereals	0⋅76 (Good)	0⋅85 (Good)	0⋅93 (Good)	<0⋅001 (Poor)	16⋅0 (Acceptable)	80⋅0	2⋅8 (Good)	0⋅56 (Moderate)	0⋅78 (Moderate)
ASF	0⋅64 (Good)	0⋅68 (Good)	0⋅77 (Good)	0⋅04 (Poor)	14⋅0 (Acceptable)	46⋅7	0⋅0 (Good)	0⋅43 (Moderate)	0⋅56 (Moderate)
Milk	0⋅88 (Good)	0⋅88 (Good)	0⋅91 (Good)	0⋅0009 (Poor)	13⋅7 (Acceptable)	75⋅2	0⋅0 (Good)	0⋅76 (Good)	0⋅90 (Excellent)
Legumes	0⋅39 (Acceptable)	0⋅38 (Acceptable)	0⋅51 (Good)	0⋅11 (Good)	20 (Acceptable)	57⋅1	3⋅8 (Good)	0⋅28 (Fair)	0⋅46 (Moderate)
Fruits	0⋅50 (Good)	0⋅48 (Acceptable)	0⋅48 (Acceptable)	0⋅004 (Poor)	33⋅5 (Poor)	43⋅8	8⋅5 (Good)	0⋅29 (Fair)	0⋅47 (Moderate)
Vegetables	0⋅37 (Acceptable)	0⋅35 (Acceptable)	0⋅43 (Acceptable)	0⋅006 (Poor)	41⋅0 (Poor)	26⋅7	6⋅7 (Good)	0⋅26 (Fair)	0⋅21 (Moderate)
Processed foods	0⋅46 (Acceptable)	0⋅65 (Good)	0⋅65 (Good)	0⋅15 (Good)	8⋅2 (Good)	40⋅9	2⋅8 (Good)	0⋅28 (Fair)	0⋅31 (Moderate)
Nutrients									
Energy	0⋅68 (Good)	–	–	0⋅002 (Poor)	13 (Acceptable)	43⋅8	2⋅8 (Good)	0⋅47 (Moderate)	0⋅62 (Moderate)
Carbohydrate	0⋅67 (Good)	0⋅68 (Good)	0⋅69 (Good)	<0⋅001 (Poor)	20 (Acceptable)	46⋅6	3⋅8 (Good)	0⋅44 (Moderate)	0⋅61 (Moderate)
Protein	0⋅70 (Good)	0⋅50 (Good)	0⋅50 (Good)	<0⋅001 (Poor)	18 (Acceptable)	47⋅6	1⋅9 (Good)	0⋅49 (Moderate)	0⋅63 (Moderate)
Fats	0⋅70 (Good)	0⋅74 (Good)	0⋅75 (Good)	0⋅25 (Good)	-2 (Good)	43⋅8	0 (Good)	0⋅48 (Moderate)	0⋅70 (Moderate)
Dietary fibre	0⋅63 (Good)	0⋅74 (Good)	0⋅74 (Good)	<0⋅001 (Poor)	27 (Poor)	49⋅5	4⋅7 (Good)	0⋅42 (Moderate)	0⋅62 (Moderate)
Iron	0⋅49 (Acceptable)	0⋅60 (Good)	0⋅60 (Good)	<0⋅001 (Poor)	24 (Poor)	40⋅9	7⋅6 (Good)	0⋅30 (Fair)	0⋅48 (Moderate)
Zinc	0⋅69 (Good)	0⋅57 (Good)	0⋅57 (Good)	<0⋅001 (Poor)	20 (Acceptable)	48⋅5	9⋅5 (Good)	0⋅46 (Moderate)	0⋅59 (Moderate)
Vitamin A	0⋅83 (Good)	0⋅46 (Acceptable)	0⋅46 (Acceptable)	0⋅002 (Poor)	17 (Acceptable)	34⋅2	6⋅6 (Good)	0⋅36 (Fair)	0⋅33 (Moderate)
Calcium	0⋅53 (Good)	0⋅84 (Good)	0⋅85 (Good)	0⋅003 (Poor)	10 (Good)	51⋅4	0⋅0 (Good)	0⋅60 (Good)	0⋅87 (Excellent)
Magnesium	0⋅60 (Good)	0⋅39 (Acceptable)	0⋅39 (Acceptable)	<0⋅001 (Poor)	23⋅7 (Poor)	47⋅6	3⋅8 (Good)	0⋅40 (Moderate)	0⋅48 (Moderate)
Thiamine	0⋅70 (Good)	0⋅56 (Good)	0⋅56 (Good)	<0⋅001 (Poor)	19 (Acceptable)	47⋅1	1⋅9 (Good)	0⋅48 (Moderate)	0⋅61 (Moderate)
Folates	0⋅55 (Good)	0⋅46 (Acceptable)	0⋅47 (Acceptable)	<0⋅001 (Poor)	22 (Poor)	41⋅9	4⋅1 (Good)	0⋅33 (Fair)	0⋅35 (Moderate)
Vitamin C	0⋅35 (Acceptable)	0⋅30 (Acceptable)	0⋅30 (Acceptable)	<0⋅001 (Poor)	31 (Poor)	47⋅6	9⋅5 (Good)	0⋅24 (Fair)	0⋅65 (Moderate)

* Coefficients are significant at *P* < 0⋅001 for all the foods, nutrients and energy.

a To de-attenutate the energy-adjusted coefficient for the within-individual variances in the intakes from the repeated recalls.

b To assess the mean difference in the intakes recorded by the tools.

c [SQFFQ − Avg. of two 24-h recalls]/Avg. of two 24-h recalls × 100.

d Ranking of the intakes measured by the SQFFQ in the same and to extreme opposite quintiles as measured by the 24-h DR.

e Cohen's weighted kappa statistic for quartiles (1 2 ji 2 jj=ðk 2 1Þ; where i and j index the rows and columns of the two ratings and k ¼ 4).

f Lin's concordance coefficient is a measure of accuracy and precision of the agreement.

The intakes of energy and the macronutrients (carbohydrate and protein) measured by the SQFFQ were significantly higher (*P* < 0⋅05) than that measured by the 24-h DRs, with an exception of fats (*P* = 0⋅25) (Tables 2 and 3). Similarly, for the key micronutrients, such as iron, zinc, vitamin A, calcium and folic acid, the intakes measured by the SQFFQ were statistically significantly higher (*P* < 0⋅05) compared with that measured by the 24-h DRs (Tables 2 and 3).

#### Test results and the assessment of the agreements of the compared methods

Table 3 presents the assessment of the validity of the SQFFQ and the interpretation of agreement of the measurements for food, energy and nutrient intakes derived from the 7-d SQFFQ and 24-h DRs. The energy-adjusted correlation coefficients were ‘good’ for the cereals (0⋅85), animal source foods (0⋅68), milk (0⋅88) and the processed foods (0⋅65); while the coefficient was ‘acceptable’ for legumes (0⋅38), vegetables (0⋅35) and the fruits (0⋅48). The de-attenuated coefficients accounting for the within-subject variations of intakes showed the improvement of the association for cereals (0⋅93), animal source foods (0⋅77), milk (0⋅91), legumes (0⋅51) and vegetables (0⋅43); while it remained unchanged for other food groups. All the coefficients were significant at *P* < 0⋅001. The energy-adjusted coefficients for the nutrients were ‘good’ for the macronutrients (0⋅50–0⋅74; *P* < 0⋅001) and for most of the micronutrients (0⋅56–0⋅84; *P* < 0⋅001); while the coefficients were ‘acceptable’ for vitamin A, magnesium, folate and vitamin C (0⋅30–0⋅46; *P* < 0⋅001). De-attenuated coefficients largely remained unchanged for all the macronutrients and micronutrients (*P* < 0⋅001). The percent difference for the measurements between the SQFFQ and the reference tool was ‘acceptable’ for most food groups (cereals, animal source foods, milk and legumes), ‘good’ for the processed foods and ‘poor’ for fruits and vegetables. Regarding the nutrients, the percent difference was ‘good’ for fats and calcium; ‘acceptable’ for energy, carbohydrates, proteins, zinc, vitamin A and thiamine and ‘poor’ for dietary fibre, iron, magnesium, folate and vitamin C.

The classification by the SQFFQ in the same quintile as measured by the 24-h DRs was seen with >40 % of the respondents with six of the seven food groups; with high proportions for cereals (80 %) and milk (75⋅2 %). The classification in the extreme opposite quintile was reported in <5 % of the respondents with six of the seven food groups. The classification in the same quintile was observed in >40⋅0–49⋅5 % of the respondents for energy and all the macronutrients and micronutrients, except for vitamin A, which was 34⋅2 %. The classification in the extreme opposite quintile was observed in 0 to <10 % of the respondents for all the nutrients. All the food groups and the nutrients were classified with a fair level of closeness, as depicted by the kappa estimates ranging from 0⋅24 to 0⋅76 (*P* < 0⋅001). Lin's concordance correlation for the absolute agreement showed that the coefficient (rho_c) was ‘excellent’ for milk (0⋅90; *P* < 0⋅001) and ‘moderate’ for other food groups (0⋅28–0⋅76; *P* < 0⋅001). The absolute agreement was ‘moderate’ for all the macronutrients and micronutrients and energy (0⋅30–0⋅70; *P* < 0⋅001), except for calcium which had an ‘excellent’ absolute agreement (0⋅87; *P* < 0⋅001).

**Fig. 2.**
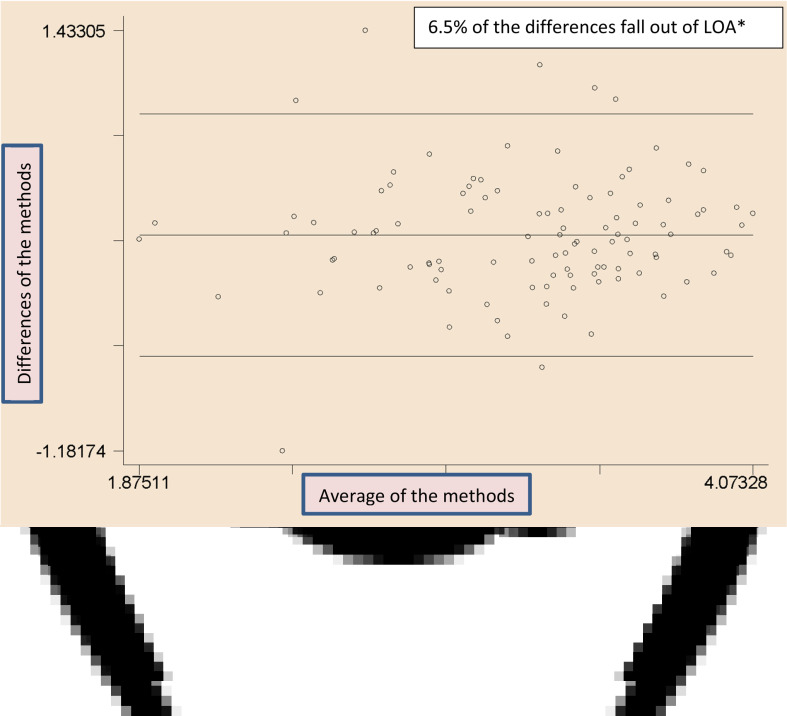
(a) Bland–Altman plots showing agreements between the SQFFQ *v*. 24-h DRs in measuring the intakes of energy. (b) Bland–Altman plots showing agreements between the SQFFQ *v*. 24-h DRs in measuring the intakes of protein. (c) Bland–Altman plots showing agreements between the SQFFQ *v*. 24-h DRs in measuring the intakes of carbohydrate. (d) Bland–Altman plots showing agreements between the SQFFQ *v.* 24-h DRs in measuring the intakes of fat. *LOA, Limits of Agreement (within ±1⋅96 sd of the mean differences between the methods).

**Fig. 3.**
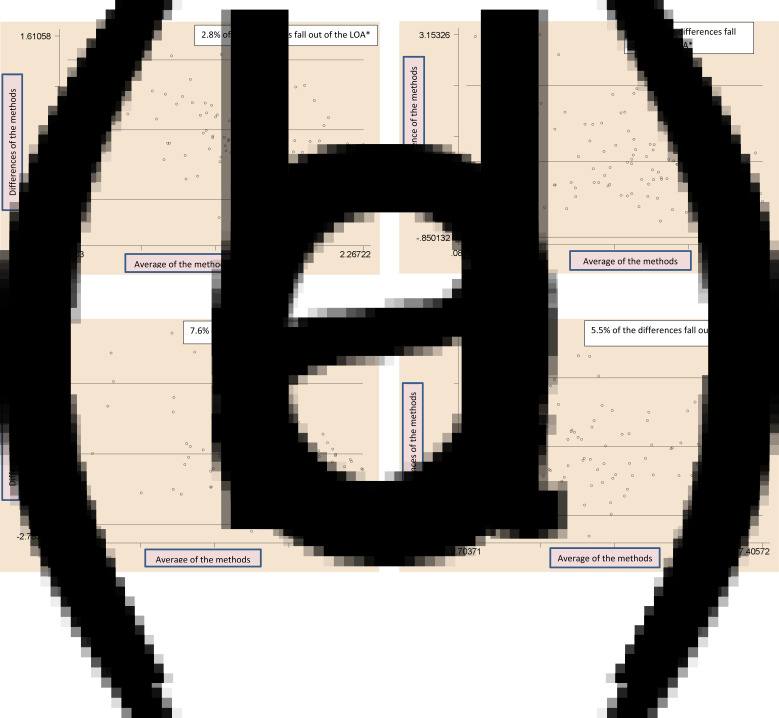
Bland–Altman plots showing agreements between the SQFFQ *v.* 24-h DRs in measuring the intakes of iron. (b) Bland–Altman plots showing agreements between the SQFFQ *v.* 24-h DRs in measuring the intakes of zinc. (c) Bland–Altman plots showing agreements between the SQFFQ *v*. 24-h DRs in measuring the intakes of vitamin A. (d) Bland–Altman plots showing agreements between the SQFFQ *v*. 24-h DRs in measuring the intakes of calcium. *LOA, Limits of Agreement (within ±1⋅96 sd of the mean differences between the methods).

The analysis of Bland–Altman plots (Figs. 2(a)–(d) and 3(a)–(d)) showed that the key macronutrient and micronutrient intakes did not present a significant proportional bias and most of the points fell within the 95 % limits of agreement. Only a few of the points fell outside the agreement limits, which were between 2⋅8 and 7⋅6 %, for all the macronutrients and micronutrients. For example, for proteins, carbohydrates and fats, the estimates were 6⋅5, 4⋅7 and 2⋅8 %, respectively. For iron, zinc and vitamin A, the estimates were 2⋅8, 3⋅8 and 7⋅6 %, respectively.

### Overall appraisal of the results

After appraisal of the domains of the assessment as per the EURRECA guidelines, the overall score of the study was 4⋅5, consistent with the rank of ‘good’ (Table 4).

**Table 4.** Evaluation of the study in the framework of the European Micronutrient recommendations aligned network of excellence (EURRECA)^(38)^a^^

Domains	Standard elements	Designated points	Elements included in the present study	Scored points	EURRECA classification
Sample and sample size	Non-homogenous sample (sex, obesity)	0⋅5	Homogenous sample	0⋅0	‘Good’
	Sample size > 100	0⋅5	Sample size > 100	0⋅5	
Statistics					
Group level	Compare/test mean or median or difference	1⋅0	Test means	1⋅0	
Correlations	Unadjusted	0⋅5			
	Energy-adjusted	1⋅0			
	De-attenuated or intra-class correlations	1⋅5	De-attenuated	1⋅5	
Agreement	Classification or Bland–Altman plots	0⋅5	Classification, Bland–Altman plots, Lin's concordance, etc.	0⋅5	
Data collection	Gathered by face-to-face interview	1⋅0	Gathered by face-to-face interview	1⋅0	
Seasonality	Considered	0⋅5	Not considered	0⋅0	
Supplements information	Included and data considered in analysis	1⋅5	Not included	0⋅0	
Total points		7⋅0		4⋅5	

a The domains of the assessment are (a) the sample and sample size, (b) statistics: group means, correlations and agreements, (c) the data collection method, (d) seasonality and (e) the inclusion of supplements.

Interpretation: (1) Very good/excellent: ≥5⋅0–7⋅0; (2) Good: 3⋅5 to <5; (3) Acceptable/reasonable: 2⋅5 to <3⋅5; (4) Poor: <2⋅5.

## Discussion

In the present study, we assessed the validity of a 7-d SQFFQ used in a randomised controlled trial to examine the efficacy of MNP supplementation in Bangladeshi rural children aged 24–59 months. Unlike the traditional way of conduction of FFQ, the 7-d SQFFQ was interviewer-administered, had an open-ended actual number of times of consumption and the reference time of food intake of 7 d. The present study compared the daily intakes of foods and nutrients measured by the 7-d SQFFQ with the average of the two 24-h DRs using a battery of statistical tests. The results showed that this SQFFQ demonstrated good/acceptable validity against the 24-h DRs for most food groups and nutrients. The classification in the extreme opposite quintile was observed in 0 to <10 % of the respondents for all the nutrients. All the food groups and the nutrients were classified with a fair level of closeness, as depicted by the kappa estimates.

Overall, the SQFFQ overestimated the intakes of the foods and nutrients compared with that estimated by the 24-h DRs. This finding is expected and consistent with other studies^(39–43)^. The possible reason for overestimated intakes is believed to be due to the fact that parents may not adequately assess the small portion sizes consumed by their children sometimes without consuming full portions, leading to the overestimation of the portion size for some foods^(39,42)^. Despite some overestimation, the SQFFQ provided acceptable estimates of the intakes in the young children with good agreement with the 24-h DRs.

There is a paucity of studies in Bangladesh which validated the FFQ with an open-ended frequency of consumption option and considered a similar comparator method as used in the present study. In an assessment of the external validity of the present SQFFQ, we compared its measured energy intake to a dietary assessment study in rural Bangladeshi children aged 24–48 months using 12-h recall and 12 h of weighing observation^(44)^. The daily intake of energy was 998⋅4 and 889 kcal by the present study and by Arsenault *et al.*^(44)^, respectively. Considering a slight mismatch of age group (24–59 months in the present study *v*. 24–48 months in the Arsenault *et al.* study) and a considerable difference in the assessment methods, the energy estimated by the present tool appears to be reasonable.

The de-attenuated correlation coefficients marked an increase from the energy-adjusted coefficients for food groups, such as cereals, animal source foods, legumes and vegetables. This implies that there is some degree of the within-subject variance of intakes measured over the repeated recalls. The within-subject variance of intake was small for rice (results not shown). The day-to-day variances in the intakes of bread (flat bread and sliced bread), which are consumed less consistently in this setting, might have contributed to some within-subject variance for the cereals group. Hence, the de-attenuation of the variance has improved the coefficient in the cereal group. Animal sourced food is expensive for rural families, and the within-subject variance of intake was common (results not shown), which led to the larger de-attenuated coefficient. Legumes and vegetables were consumed sparsely in this age group, with some within-subject variances over the 24-h DRs and, therefore, resulting in larger de-attenuated coefficients. Regarding the nutrients, there was hardly any difference between the energy-adjusted and the de-attenuated coefficients. This is difficult to explain; however, we observed that the within-subject variances of the intakes of the nutrients over the two 24-h DRs were very small leading to the negligible within-subject to between-subject ratios (results not shown). The underlying reason for this could be the distribution of the nutrients in the foods commonly consumed was largely homogenous, and the children's dietary pattern was less-diversified throughout assessments.

Consistent with the study of Lovell *et al.*^(38)^, we observed an increasing magnitude of the coefficient of the association of the SQFFQ and the reference method, as the frequency of consumption increased. In this setting, the consumption of cereals, e.g. rice, and the consumption of milk were frequent and consistent, and very large energy-adjusted and de-attenuated correlation coefficients were observed regarding those foods. However, much smaller de-attenuated coefficients were observed for vegetables and fruits, since the intakes of those foods were less consistent. The smaller coefficients with the episodically consumed food items are consistent with another study^(45)^ due to the high day-to-day variability of the intakes.

A ‘poor’ percent difference between the intake estimates measured by the methods was observed for some of the nutrients, such as dietary fibre, iron, magnesium, folate and vitamin C. Of this methodological difference of estimates, iron and magnesium were marginally outside the 20 % cut-off (i.e. the magnitude of difference above which suggesting a ‘poor’ agreement). However, for vitamin C (31 %) and dietary fibre (27 %), the difference was on the higher side. One of the explanations for this is that, the children in this age and setting are less likely to consume vegetables and fruits consistently. This is supported by the observation that the SDs for the mean intakes were larger for the 24-h DRs compared with the SQFFQ regarding vegetables, fruits and vitamin C. This is the suggestive of infrequent consumption of these food items in the children. Hence, while the SQFFQ might have captured the intakes but the 24-h DRs failed, leading to the widening of differences between the methods.

The correlation coefficients and the level of agreement of the present SQFFQ with the reference method were larger than the agreements observed in other FFQs validated in Bangladeshi populations^(10,11)^, and it was larger than the median coefficients reported in a systematic review of FFQ validation studies^(46)^. The possible reasons for the difference in the coefficient estimates are the tool itself, the reference tool and the methods of administration. Lin *et al.*^(10)^ used an interviewer-administered dish-based SQFFQ with up to a 1-year reference time, and the respondent-administered FDs was the reference method. Chen *et al.*^(11)^ used an interviewer-administered FFQ with open-ended consumption options but with a 1-year reference time and the interviewer-administered two 7-d FDs as the reference method. The reason for the larger coefficient estimates and a higher level of agreement observed in the present study than the above-referred studies is perhaps the short reference time. With the short time span, the respondents could report the actual intake with higher precision, rather than reporting habitual consumptions over a long reference time recorded in the other studies. The other reason for the larger coefficients observed in the present SQFFQ is the time of administration of the 24-h DRs. After taking the SQFFQ, the two non-consecutive recalls were completed within 2 weeks. Within this short interval, the pattern of intake was largely unchanged. Both the test and the reference methods were interviewer-administered, who could record the amount of intakes with good precision, which might have contributed further to the high levels of agreement.

The present study findings have implications on the dietary intake assessment of the children recruited in a trial assessing the effect of an MNP supplementation on haemoglobin and iron parameters. The nutrients of particular interest to the trial were iron, zinc, vitamin A, vitamin C and folic acid. The de-attenuated correlation coefficients were consistent with ‘good’ to ‘acceptable’ for the nutrients. Lin's concordance of absolute agreement between the methods for these nutrients was ‘moderate’ justifying the usage of the SQFFQ for a valid estimate of the children's intake of the micronutrients in the concerned trial.

## Strength and limitations

The strength of our SQFFQ is the fact that it recorded the actual number of intakes of foods over 7 d preceding the interviews, unlike the habitual frequency of consumption captured in other types of FFQs. Cade *et al.*^(9)^ reported that the commonly employed statistical methods in the SQFFQ validation studies are correlation, percent difference, cross-classification, kappa estimates of agreement and Bland–Altman plots. A recent review by Lovell *et al.*^(38)^ reported that the mean comparison, correlations, cross-classifications and kappa statistics are commonly reported statistical workup in dietary validation studies. Lin *et al.*^(10)^ in the validation of a dish-based SQFFQ in the Bangladesh context have performed all the above-stated tests. The present study in addition to the above-stated statistical tests reported the concordance agreement; hence, the robustness of the assessment is the strength of the study. The method will be useful in the epidemiological dietary assessment in preschool children by providing a convenient alternative to the standard methods, e.g. 24-h DRs, which typically needs multiple non-consecutive day administrations posing logistical challenges. The tool is generalisable for the dietary assessment of Bangladeshi children aged 2–5 years, since the foods of the FFQ are derived from a nationally representative dietary assessment survey as the foods commonly consumed.

However, the present study has some limitations. Due to logistical difficulties, we could not repeat the SQFFQ. The single-time administration of the method failed to test the reproducibility and seasonality. Despite the SQFFQ captured the most commonly consumed foods in a largely homogenous longitudinal intake pattern of the rural Bangladeshi setting; not testing the seasonality may compromise its long-term validity for some micronutrients, such as vitamin A, which is consumed in higher amount in the summer fruit season. Secondly, we used 24-h DR as the reference method. Both 24-h DRs and FFQs are prone to measurement error associated with the recall bias and the awareness of portion size. Errors associated with these methods are not mutually independent, and the correlation coefficients might have been overestimated^(15)^. Thirdly, data on continuing breastfeeding were not gathered due to difficulties in measuring the breast milk quantity^(47)^. However, as breastfeeding was not recorded by either of the competing methods, it is unlikely to affect the comparability about the relative validity. Nonetheless, not inquiring about the data constitutes a limitation. Fourthly, the possible recall bias for accommodating both the methods – SQFFQ and the first 24-h recall in the same interview – may not be ruled out. We attempted to minimise it by orienting the interviewers about such possibility and conduct the communication to allay this as much as possible. The methods being administered by the interviewers and not being self-reported aided in minimising the issue. Traditionally, FFQs tend to overestimate the measure when compared with 24-h recalls^(39–43)^. The estimates of the present FFQ reported somewhat higher values than that reported by the recalls; hence, it was consistent with the general trend of the relative measure between these methods.

Fifthly, for most of the non-processed foods, the nutrient intake was calculated as per its content in 100 g of raw food as per the updated FCT of Bangladesh. Some nutrients, such as folate and vitamin C, are lost to some extent during the cooking process. Not accounting for such losses is a limitation of the study. Lastly, the FFQ had the limitation to assess vitamin C intake as the low agreement levels were observed in terms of kappa statistics, cross-classification and percent differences. Fruits and vegetables and the rich sources of vitamin C are eaten sparsely by the children in this setting. Hence, validating with a low number (*n* 2) of the recalls might have led to the recall-days when the child did not take a vitamin C-rich food, and thus, the poor agreement is expected.

The study performed well among the FFQ validation studies in light of the assessment over some standard metric parameters^(38)^, and ranked high among the studies tagged as ‘good’ classification. The study did not consider the intake of supplements; thus, it just fell short off the ‘excellent’ ranking according to Lovell's appraisal^(38)^. However, the usage of supplements among preschool age rural Bangladeshi children is rare.

## Conclusion

In conclusion, the interviewer-administered, 7-d SQFFQ with an open-ended actual number of times of intake is a valid tool in assessing the food and nutrient intakes in 24–59 months old Bangladeshi children. The tool can be used for assessing children's short-term intake of food in the epidemiological studies in Bangladesh, where the respondent's literacy is suboptimum.

## References

[1] Cagliari MPP, Paiva AA, Queiroz D, (2009) Food consumption, anthropometry and morbidity in preschool children from public day care centers of Campina Grande, Paraíba. Nutrire: rev. Soc. Bras. Alim. Nutr. 34, 29–43.

[2] Menezes MC, Horta PM, Santos LC, (2011) Avaliação do consumoalimentar e de nutrientes no contexto da atençãoprimária à saúde. Ceres 6, 175–190.

[3] National Cancer Institute – Dietary Assessment Primer. Food Frequency Questionnaire at a Glance. https://dietassessmentprimer.cancer.gov/profiles/questionnaire/ (accessed 28 August 2020).

[4] Tang Y, Liu Y, Xu L, (2015). Validity and reproducibility of a revised semi-quantitative food frequency questionnaire (SQFFQ) for women of age-group 12–44 years in Chengdu. J Health Popul Nutr 33, 50–59. 25995721 PMC4438648

[5] Data4diets. International Dietary Data Expansion Project (INDDEX). Food Frequency Questionnaires (FFQ). https://inddex.nutrition.tufts.edu/data4diets/data-source/food-frequency-questionnaires-ffq (accessed 28 August 2020).

[6] Naska A, Lagiou A & Lagiou P (2017) Dietary assessment methods in epidemiological research: current state of the art and future prospects. F1000Res 6, 926. 28690835 10.12688/f1000research.10703.1 PMC5482335

[7] Rahman S, Ahmed T, Rahman AS, (2016) Determinants of iron status and Hb in the Bangladesh population: the role of groundwater iron. Public Health Nutr 19, 1862–1874. 26818180 10.1017/S1368980015003651 PMC10270950

[8] Iqbal MS, Rahman S, Haque MA, (2019) Lower intakes of protein, carbohydrate, and energy are associated with increased global DNA methylation in 2- to 3-year-old urban slum children in Bangladesh. Matern Child Nutr 15, e12815. 30903804 10.1111/mcn.12815 PMC7198919

[9] Cade J, Thompson R, Burley V, (2002) Development, validation and utilisation of food-frequency questionnaires – a review. Public Health Nutr 5, 567–587. 12186666 10.1079/PHN2001318

[10] Lin PD, Bromage S, Mostofa MG, (2017) Validation of a dish-based semi-quantitative food questionnaire in rural Bangladesh. Nutrients 9, 49. 10.3390/nu9010049 PMC5295093 28075369

[11] Chen Y, Ahsan H, Parvez F, (2004) Validity of a food-frequency questionnaire for a large prospective cohort study in Bangladesh. Br J Nutr 92, 851–859. 15533275 10.1079/bjn20041277

[12] United States Department of Agriculture (2002) Agricultural Research Service Nutrient Data Laboratory Home Page. USDA Nutrient Database for Standard Reference, Release 15. http://www.nal.usda.go/ni/oodcom/at/R1/r15.html

[13] Gopalan C, Rama Sastri BV & Balasubramanian SC (1989) Nutritive Value of Indian Foods. Hyderabad, India: National Institute of Nutrition, Indian Council of Medical Research.

[14] Willett WC (editor) (1998) Nutritional Epidemiology, 2nd ed. New York, NY: Oxford University Press.

[15] Willett W (2013) Nutritional Epidemiology: Monographs in Epidemiology and Biostatistics, 3rd ed. New York: Oxford University Press.

[16] Institute of Public Health Nutrition, UNICEF Bangladesh, ICDDR, B, (2013) National Micronutrient Survey: 2011–12. Final Report. Dhaka: Institute of Public Health Nutrition, United Nations Children's Fund, Bangladesh, ICDDR, B and GAIN.

[17] Islam SN, Khan MNI & Akhtaruzzaman M (2010) A Food Composition Database for Bangladesh with Special Reference to Selected Ethnic Foods. Final Report. NFPCSP, GoB.

[18] Institute of Nutrition, IN (1973) Nutritive Values of Some Common Food Stuff. Reports of the Research Activities of the Institute of Nutrition, University of Dacca, Institute of Nutrition (now INFS) DU, Dhaka.

[19] Shaheen N, Rahim ATMA, Mohiduzzaman M, (2014) Food Composition Table for Bangladesh. Dhaka: Institute of Nutrition and Food Science Centre for Advanced Research in Sciences, University of Dhaka.

[20] United States Department of Agriculture, Agricultural Research Service. https://www.ars.usda.gov/northeast-area/beltsville-md-bhnrc/beltsville-human-nutrition-research-center/food-surveys-research-group/docs/main-service-page/#

[21] FAO (2018) Dietary Assessment: A Resource Guide to Method Selection and Application in Low Resource Settings. Rome.

[22] Bingham SA & Day NE (1997) Using biochemical markers to assess the validity of prospective dietary assessment methods and the effect of energy adjustment. Am J Clin Nutr 65, 1130S–1137S. 9094909 10.1093/ajcn/65.4.1130S

[23] Liu K, Stamler J, Dyer A, (1978) Statistical methods to assess and minimize the role of intraindividual variability in obscuring the relationship between dietary lipids and serum cholesterol. J Chronic Dis 31, 399–418. 711832 10.1016/0021-9681(78)90004-8

[24] Beaton GH, Milner J, Corey P, (1979) Sources of variance in 24-hour dietary recall data: implications for nutrition study design and interpretation. Am J Clin Nutr 32, 2546–2549. 506977 10.1093/ajcn/32.12.2546

[25] Kuehneman T, Stanek K, Eskridge K, (1994) Comparability of four methods for estimating portion sizes during a food frequency interview with caregivers of young children. J Am Diet Assoc 94, 548–551. 8176133 10.1016/0002-8223(94)90222-4

[26] Venter CS, MacIntyre UE & Vorster HH (2000) The development and testing of a food portion photograph book for use in an African population. J Hum Nutr Diet 13, 205–218. 12383127 10.1046/j.1365-277x.2000.00228.x

[27] Nelson M (1997) The validation of dietary assessment. In Design Concepts in Nutritional Epidemiology, 2nd ed., pp. 241–272 [BM Margetts and M Nelson, editors]. New York: Oxford University Press.

[28] Gibson RS (2005) Principles of Nutritional Assessment. Oxford, UK: Oxford University Press.

[29] Dehghan M, Martinez S, Zhang X, (2013) Relative validity of an FFQ to estimate daily food and nutrient intakes for Chilean adults. Public Health Nutr 16, 1782–1788. 22995762 10.1017/S1368980012004107 PMC10271634

[30] Pedro MV, Ross A, Wynn E, (2011) Reproducibility and relative validity of a food-frequency questionnaire for French-speaking Swiss adults. Food Nutr Res 55, 5905. 10.3402/fnr.v55i0.5905 PMC3091846 21562629

[31] Lin LI (1989) A concordance correlation coefficient to evaluate reproducibility. Biometrics 45, 25568. 2720055

[32] Bland JM & Altman DG (1986) Statistical methods for assessing agreement between two methods of clinical measurement. Lancet 1, 307–310. 2868172

[33] Araujo MC, Yokoo EM & Alves-Pereira R (2010) Validation and calibration of a semi-quantitative food frequency questionnaire designed for adolescents. J Am Diet Assoc 110, 1170–1177. 20656092 10.1016/j.jada.2010.05.008

[34] Giavarina D (2015) Understanding Bland–Altman analysis. Biochem Med (Zagreb) 25, 141–151. 26110027 10.11613/BM.2015.015 PMC4470095

[35] Masson L, McNeill G, Tomany J, (2003) Statistical approaches for assessing the relative validity of a food-frequency questionnaire: use of correlation coefficients and the kappa statistic. Public Health Nutr 6, 313–321. 12740081 10.1079/PHN2002429

[36] Lombard MJ, Steyn NP, Charlton KE, (2015) Application and interpretation of multiple statistical tests to evaluate validity of dietary intake assessment methods. Nutr J 14, Article number: 40. doi: 10.1186/s12937-015-0027-y. 10.1186/s12937-015-0027-y PMC4471918 25897837

[37] Altman DG (1991) Practical Statistics for Medical Research. London: Chapman and Hall.

[38] Lovell A, Bulloch R, Wall CR, (2017) Quality of food-frequency questionnaire validation studies in the dietary assessment of children aged 12 to 36 months: a systematic literature review. J Nutr Sci 6, e16, page 1 of 12. 28630693 10.1017/jns.2017.12 PMC5468742

[39] Andersen LF, Lande B, Arsky GH, (2003) Validation of a semi-quantitative food-frequency questionnaire used among 12-month-old Norwegian infants. Eur J Clin Nutr 57, 881–888. 12879081 10.1038/sj.ejcn.1601621

[40] Andersen L, Lande B, Trygg K, (2004) Validation of a semi-quantitative food-frequency questionnaire used among 2-year-old Norwegian children. Public Health Nutr 7, 757–764. 15369614 10.1079/phn2004613

[41] Blum RE, Wei EK, Rockett HR, (1999) Validation of a food frequency questionnaire in native American and Caucasian children 1 to 5 years of age. Matern Child Health J 3, 167–172. 10746756 10.1023/a:1022350023163

[42] Parrish LA, Marshall JA, Krebs NF, (2003) Validation of a food frequency questionnaire in preschool children. Epidemiology 14, 213–217. 12606888 10.1097/01.EDE.0000041256.12192.23

[43] Marriott LD, Inskip HM, Borland SE, (2009) What do babies eat? Evaluation of a food frequency questionnaire to assess the diets of infants aged 12 months. Public Health Nutr 12, 967–972. 18702837 10.1017/S1368980008003388

[44] Arsenault JE, Yakes EA, Hossain MB, (2010) The current high prevalence of dietary zinc inadequacy among children and women in rural Bangladesh could be substantially ameliorated by zinc biofortification of rice. J Nutr 140, 1683–1690. 20668253 10.3945/jn.110.123059

[45] Bel-Serrat S, Mouratidou T & Pala V (2014) Relative validity of the children's eating habits questionnaire-food frequency section among young European children: the IDEFICS study. Public Health Nutr 17, 266–276. 23286734 10.1017/S1368980012005368 PMC10282422

[46] Cade JE, Burley VJ, Warm DL, (2004) Food-frequency questionnaires: a review of their design, validation and utilization. Nutr Res Rev 17, 5–22. 19079912 10.1079/NRR200370

[47] Albernaz E, Haisma H & Victora C (2000) Measurements of Breast Milk Intake in Exclusively or Predominantly Breastfed Infants, and the Impact of Lactation Counseling. Universidade Federal de Pelotas, Departamento de Medicina Social. https://inis.iaea.org/collection/NCLCollectionStore/_Public/31/053/31053126.pdf (accessed 2 March 2021).

